# Comparative transcriptome analysis reveals differentially expressed genes associated with sex expression in garden asparagus (*Asparagus officinalis*)

**DOI:** 10.1186/s12870-017-1091-6

**Published:** 2017-08-22

**Authors:** Shu-Fen Li, Guo-Jun Zhang, Xue-Jin Zhang, Jin-Hong Yuan, Chuan-Liang Deng, Wu-Jun Gao

**Affiliations:** 1 College of Life Sciences, Henan Normal University, Xinxiang, 453007 China; 2 School of Basic Medical Sciences, Xinxiang Medical University, Xinxiang, 453003 China

**Keywords:** *Asparagus officinalis*, Sex-biased gene expression, Sex determination, Sex differentiation, Sex expression, Transcriptome sequencing, Transcription factor

## Abstract

**Background:**

Garden asparagus (*Asparagus officinalis*) is a highly valuable vegetable crop of commercial and nutritional interest. It is also commonly used to investigate the mechanisms of sex determination and differentiation in plants. However, the sex expression mechanisms in asparagus remain poorly understood.

**Results:**

De novo transcriptome sequencing via Illumina paired-end sequencing revealed more than 26 billion bases of high-quality sequence data from male and female asparagus flower buds. A total of 72,626 unigenes with an average length of 979 bp were assembled. In comparative transcriptome analysis, 4876 differentially expressed genes (DEGs) were identified in the possible sex-determining stage of female and male/supermale flower buds. Of these DEGs, 433, including 285 male/supermale-biased and 149 female-biased genes, were annotated as flower related. Of the male/supermale-biased flower-related genes, 102 were probably involved in anther development. In addition, 43 DEGs implicated in hormone response and biosynthesis putatively associated with sex expression and reproduction were discovered. Moreover, 128 transcription factor (TF)-related genes belonging to various families were found to be differentially expressed, and this finding implied the essential roles of TF in sex determination or differentiation in asparagus. Correlation analysis indicated that miRNA-DEG pairs were also implicated in asparagus sexual development.

**Conclusions:**

Our study identified a large number of DEGs involved in the sex expression and reproduction of asparagus, including known genes participating in plant reproduction, plant hormone signaling, TF encoding, and genes with unclear functions. We also found that miRNAs might be involved in the sex differentiation process. Our study could provide a valuable basis for further investigations on the regulatory networks of sex determination and differentiation in asparagus and facilitate further genetic and genomic studies on this dioecious species.

**Electronic supplementary material:**

The online version of this article (doi:10.1186/s12870-017-1091-6) contains supplementary material, which is available to authorized users.

## Background

Dioecious system is the most common rule in animals, but the majority of flowering plants are cosexuals, e.g., individual plants are composed of bisexual flowers with both sexual functions. Approximately 6% of angiosperm species are dioecious plants with unisexual flowers on distinct individuals, that is, staminate flowers on males and pistillate flowers on females [1]. Diverse sex determination mechanisms in plants range from XY sex chromosomes to a completely autosomal determination system [2]. In addition, dioecy in plants has independently evolved many times. However, dioecious plants are usually derived from the lineage of hermaphrodite plants. The determination and differentiation of different sexes are genetically controlled by a pair of sex chromosomes carrying sex-determining genes [3]. At least two dominant genes, namely, one stamen-promoting gene and one gynoecium-suppressing gene, are linked in sex chromosomes [4]. Although many efforts have been made to unravel the sex-determining regulatory mechanism in recent years, only one confirmed sex-determining gene in dioecious plants has been reported in *Diospyros lotus* [5]. The mechanism and genes responsible for sex determination in various dioecious plants, even in species with sequenced genomes, remain unclear mainly because the non-recombining region of the Y/W chromosome, where the sex-determining gene is located, is usually occupied by repetitive sequences [6].

Unlike animals, whose germ cell lines are differentiated in early development stages, plants, including dioecious species, do not possess a distinct germ cell line [7]. In plants, totipotent meristematic cells usually experience a long vegetative period and then undergo the reproductive stage to form flowers, which are complex sexual organs [8]. The development and maintenance of sex-specific phenotypes, especially male and female flowers in different individuals, are under a series of metabolic pathways and regulatory genetic networks, in which various genes, transcription factors (TFs), and other regulators, such as microRNAs (miRNAs), are involved [9–11]. Similar to those in mammals [12], downstream metabolic pathways and genetic networks essential for sex differentiation in plants may be controlled by upstream sex-determining genes [5]. However, the mechanisms of sex determination and differentiation in plants are poorly understood.

Garden asparagus (*Asparagus officinalis*), belonging to the family Liliaceae, is an important dioecious vegetable crop cultivated worldwide. It contains a haploid genome size of 1308 Mb and 2*n* = 2*×* = 20 chromosomes [13]. Asparagus is also a good species for sex determination mechanistic studies because of its strict genotypic sex-determining fashion, stable sex phenotypes, and viable YY genotype individuals (supermales) [14]. Sex dimorphism in asparagus is determined by an *M* locus on the homomorphic sex chromosome L5. In particular, *MM*, *Mm*, and *mm* represent supermale, male, and female, respectively [14, 15]. Although various molecular markers linked to the *M* locus have been identified [16–18], the *M* gene has yet to be isolated and characterized. Recently, Murase et al. identified a male-specific gene *MSE1*, which plays an important role in sex determination in asparagus [19]. However, whether this gene is the exact sex-determining gene *M* should be further investigated. Various datasets and massive analyses, including EST, transcriptome, and miRNA, provide useful resources for studies on complex processes implicated in sex determination and differentiation [6, 20, 21], but genes implicated in sex determination and differentiation in asparagus remain poorly elucidated.

With advancements in next-generation sequencing technologies, transcriptome sequencing has emerged as an invaluable tool for gene discovery and related pathway enrichment. This technique has been applied to identify candidate genes underlying the traits of interest in plants [22, 23]. More importantly, several studies have been conducted to detect DEGs of different sex types, such as those in *Salix suchowensis* [24], *Carica papaya* [25], *Pelteobagrus fulvidraco* [26], and so on. Recently, Harkess et al. (2015) conducted transcriptome sequencing in male and female asparagus spear tips and identified numerous genes involved in male and female gametophyte development. Although they provided useful information regarding anther and gynoecium developmental pathways, they analyzed data from asparagus spear tips, which include both vegetative and reproductive organs. They also proposed that studying sex-biased expression in flower buds and flower bud primordia is more informative than investigating this phenomenon in spear tips [6]. In our study, flower buds from male and female asparagus were subjected to a comparative high-throughput transcriptome sequencing by using an Illumina Highseq 2500 sequencing platform to determine their corresponding DEGs. However, the supermale line was not represented. For better elucidating the sex determination and differentiation mechanisms between male and female asparagus, we connected our sequencing data with previously published data by utilizing the supermale and female flower buds of asparagus [6]. miRNA-DEG correlation analysis was also performed to identify key regulatory miRNA-targeted genes involved in sex determination or sex differentiation in asparagus.

## Results

### Sequencing and de novo transcriptome assembly of asparagus flower buds

We performed Illumina sequencing on the male and female flower bud samples, and each sample was prepared in two replicates. A total of 210,849,964 raw sequencing reads were generated from the asparagus male and female flower buds. After adapter sequences, ambiguous nucleotides, low-quality sequences, and all possible contaminations were removed, 205,739,016 clean reads remained. Of these reads, 107,013,940 were from female flower buds and 98,725,076 were from male flower buds (Table 1). To improve the quality of the assembly, we combined our sequencing data with previously reported data of the female and supermale flower buds [6] for sequence assembly. The assembly of these high-quality reads resulted in 72,626 unigenes with an N50 length of 1569 bp and an average length of 979 bp. The shortest and longest unigenes were 201 and 15,615 bp, respectively. Moreover, 34,995 (48.2%) unigenes were greater than 500 bp and 19,967 (27.5%) unigenes were larger than 1000 bp (Additional file 1). The BUSCO assessment indicated that 83.2% of the plant orthologs were covered by the assembled unigenes (Additional file 2). The score for the assemblies evaluated by Transrate was 0.242, which is above the threshold Transrate score of 0.22. These results suggested that we generated a transcriptome of asparagus flower buds with high quality.

**Table 1:** Summary of sequencing data generated and analyzed in this study

Sample	Raw Reads	Clean Reads	Clean Bases	Error (%)	GC Content(%)	Mapping ratio (%)
Ao_F1	60,940,662	59,418,208	7.42 G	0.03	42.70	84.79
Ao_F2	48,879,300	47,595,732	5.95 G	0.03	42.57	84.82
Ao_M1	53,711,620	52,492,766	6.56 G	0.03	45.87	85.20
Ao_M2	47,318,382	46,232,310	5.78 G	0.04	45.75	85.20
Ao_Female le	66,295,580	62,060,676	6.27 G	0.03	45.56	85.29
Ao_Smale	60,744,912	56,975,782	5.75 G	0.04	46.97	85.15

Ao_F and Ao_M: Female and male asparagus flower buds used in sequencing in this study

Ao_F1, Ao_F2 and Ao_M1, Ao_M2: Two replicates of female and male asparagus flower buds, respectively

Ao_Female and Ao_Smale: Female and Supermale asparagus flower buds at pre-meiotic stage used in sequencing in a previous study [6]

### Functional annotation and classification of the asparagus transcriptome

**Fig. 1:**
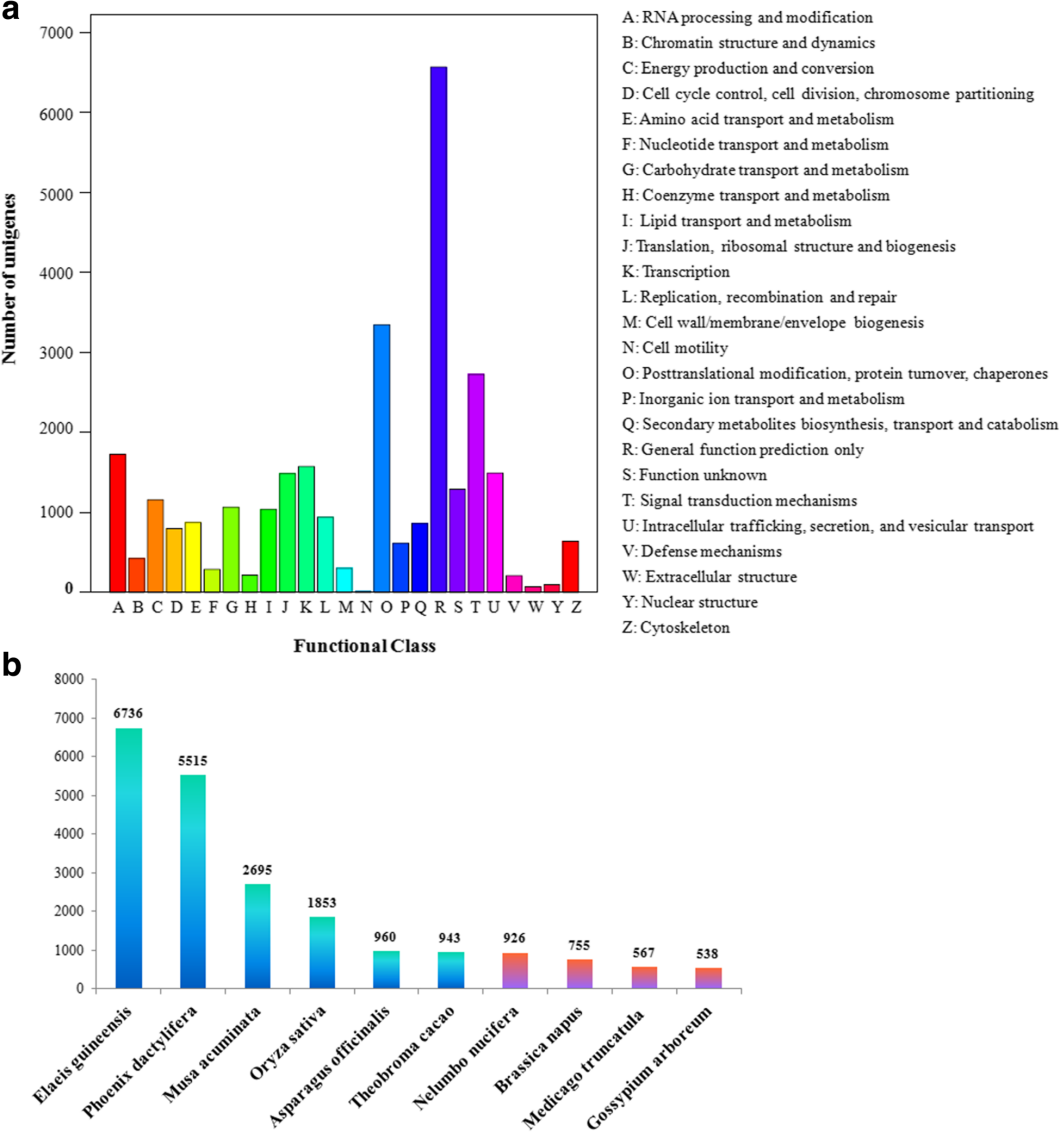
Transcriptome annotation of asparagus flower buds. **a** Transcriptome annotation of asparagus flower buds unigenes against COGs database. **b** Species distribution of the transcriptome annotation of asparagus flower buds. Only the top ten species are listed. Monocotyledon plants are shown in blue, while dicotyledon plants are shown in purple

The assembled unigenes were annotated by BLAST searching against four public databases, namely, Nr, Swiss-Prot, COG, and KEGG. The results revealed approximately 40.96%, 29.39%, 24.75%, and 16.07% unigene annotations in these databases, respectively (Table 2). In total, 29,967 (41.26%) unigenes were annotated successfully in at least one of the public protein databases (Table 2, Additional file 3). For COG annotation, 17,973 unigenes were assigned to COG classification and divided into 25 categories (Fig. 1a). The “general functional prediction only” (6564, 36.52%) was the largest group, followed by “posttranslational modification, protein turnover, chaperones” (3344, 18.61%), “signal transduction mechanisms” (2727, 15.17%), and “RNA processing and modification” (1725, 9.60%). Very few unigenes were assigned to “cell motility” (12, 0.07%).

**Table 2:** Functional annotation of assembled asparagus unigenes

Public database	Number of unigenes	Percentage (%)
Nr	29,748	40.96
SwissProt	21,344	29.39
COG	17,973	24.75
KEGG	11,669	16.07
Annotated in at least one Database	29,967	41.26
Total unigenes	72,626	100

Species distribution analysis showed that the unigenes of asparagus matched those of various plant species (Fig. 1b). Among numerous plant species, *Elaeis guineensis* (6736), followed by *Phoenix dactylifera* (5515), *Musa acuminata* (2695), and *Oryza sativa* (1853), contained sequences with the highest hits with the unigenes of asparagus. Therefore, most of the annotated unigenes exhibited high hits with the sequences from monocotyledon plants, and this finding is consistent with the fact that asparagus is a monocotyledon species.

### Comparison of transcriptomes between male and female asparagus flower buds

**Fig. 2:**
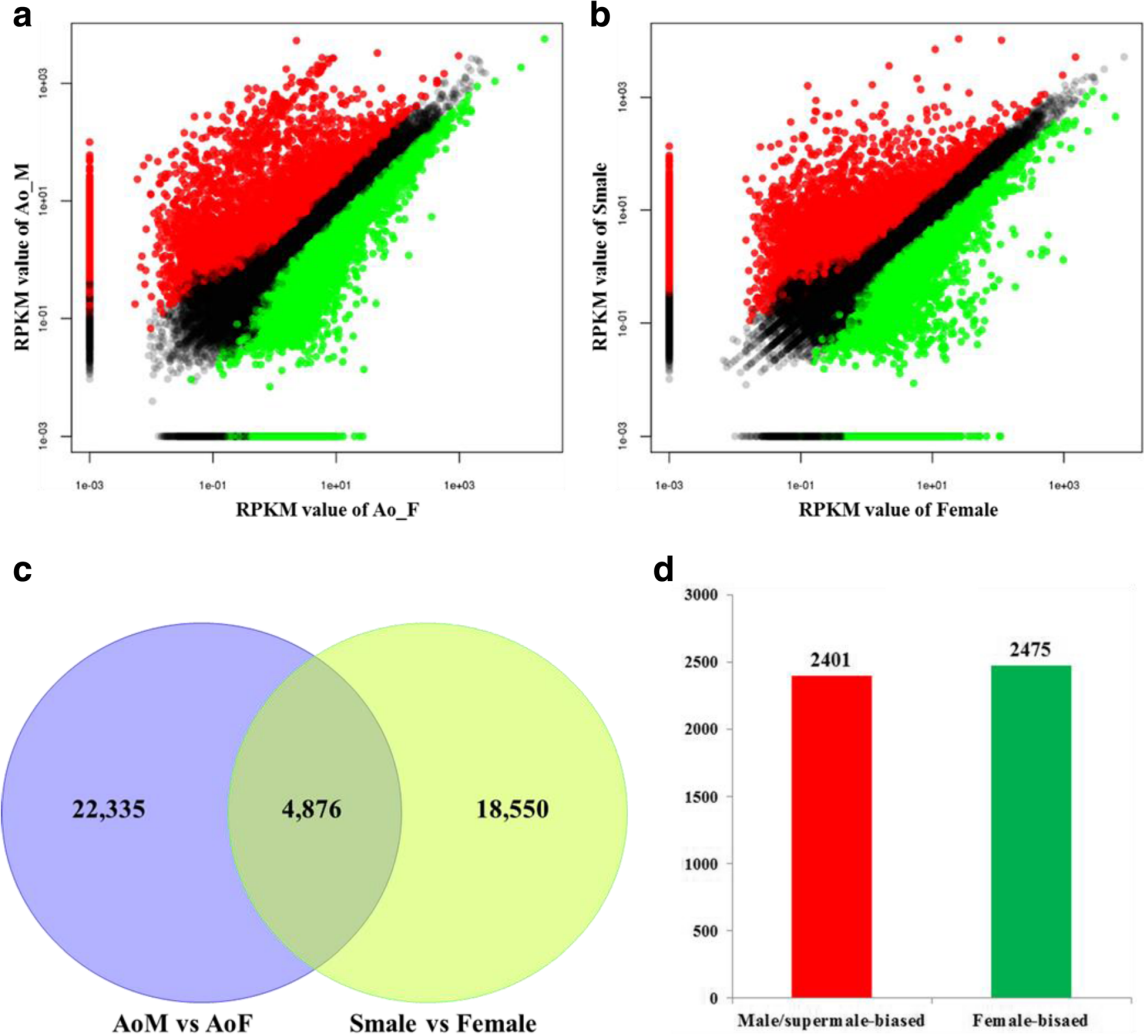
Comparisons of gene expression between different sexual types of asparagus. **a** Gene expression comparison between male and female plants. **b** Gene expression comparison between supermale and female plants. **c** Shared differentially expressed genes between male vs. female and supermale vs. female flower buds. **d** Numbers of shared male/supermale-biased and female-biased genes

Digital expression profiling analysis showed that 22,335 genes were differentially expressed in male and female flower buds (Fig. 2a), while 18,550 genes were differentially expressed in supermale and female flower buds (Fig. 2b). A specific gene responsible for sex determination or sex differentiation should exhibit a similar expression pattern in male and supermale plants versus female plants. Thus, we identified the genes with similar expression patterns between male/supermale and female plants for further analysis. A total of 4876 genes were differentially expressed between the male/supermale and female flower buds (Fig. 2c). Of these genes, 2401 exhibited a significantly higher expression in male/supermale flower buds than in female flower buds and 2475 exhibited a biased expression in female flower buds (Fig. 2d, Additional file 4).

Among these DEGs, sex-specific genes were detected to search for potential genes that are present or absent in different sex types. The following criteria were used: DEGs contained an estimated abundance of absolutely zero counts in one sex type but showed a certain expression in the other type (read per kilobase per million, RPKM >0.03). Finally, 166 unigenes were identified as sex-specific genes. Of these unigenes, 120 were male/supermale specific and 46 were female specific (Additional file 5). Therefore, the majority of the genes exhibited a male/supermale-specific expression pattern.

**Fig. 3:**
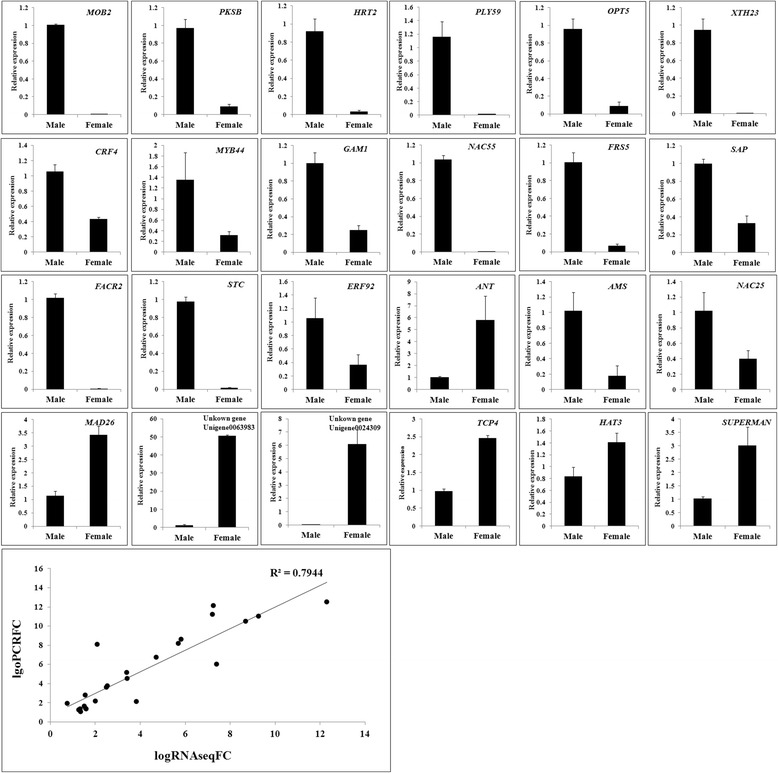
Quantitative RT-PCR validation of differentially expressed unigenes between male and female asparagus flower buds

To confirm the reliability of the RNA-Seq data, we examined the transcriptional level of 24 unigenes selected semi-randomly through real-time quantitative PCR (Fig. 3). We randomly selected 18 male-biased genes and 6 female-biased genes because male-biased genes are considered more informative than female-biased ones. Although the fold-change value was different between RT-PCR and RNAseq, the expression patterns of the unigenes were consistent with the RNA-Seq data (*R* ^2^ = 0.7944).

The DEGs between male/supermale and female flower buds were then used as input to perform Gene Ontology (GO) annotation, and GO terms were classified into three categories: cellular component, molecular function, and biological process. A total of 1619, 1060, and 2193 male/supermale-biased DEGs were respectively assigned to these categories, whereas 1118, 642, and 1402 female-biased DEGs were respectively designated to these categories (Additional file 6).

**Fig. 4:**
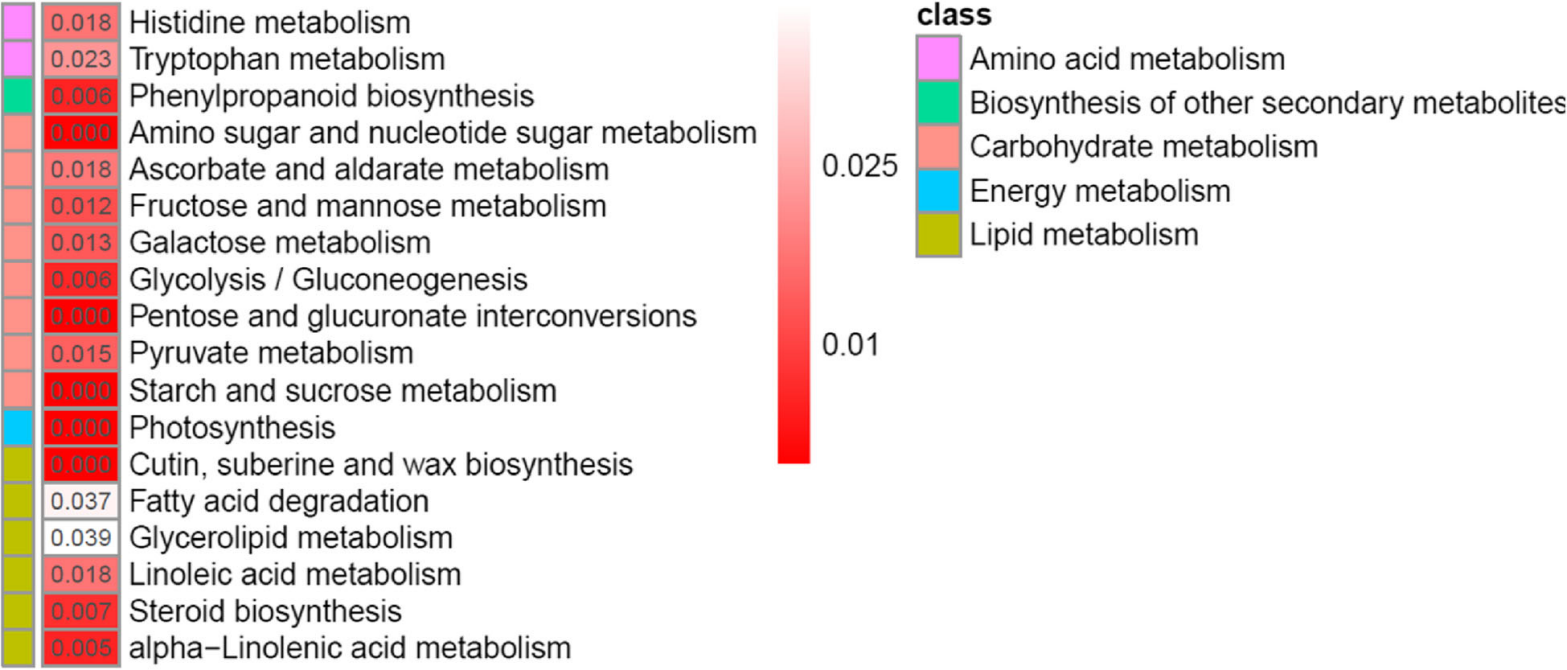
Heatmap representation of significant functional groups of differentially expressed genes between male/supermale and female flower buds. Colors show the *p*-value, with dark red representing low *p*-value and light red representing high *p*-value

To better annotate the DEGs, we collected all *Arabidopsis* genes with functions associated with plant reproduction and flower development. BLAST analyses suggested that 5809 unigenes were putatively homologous to *Arabidopsis* gene products. Of these unigenes, 433, including 284 male/supermale-biased genes and 149 female-biased genes, were DEGs between male/supermale and female flower buds (Fig. 4, Additional file 7). Of the sequences annotated as reproduction and flower development related, 102 unigenes were effectively annotated as anther-related genes or male-sterile mutant-related genes (Fig. 4). The genes with male/supermale-biased expression in asparagus flower buds included the homologs of *Aborted Microspore*s (*AMS*), *Polygalacturonase 2* (*PG2*), *Microspore-specific promoter 2* (*MSP2*), and other anther-related genes. In particular, 11 genes with male/supermale-biased expression were involved in pollen wall formation processes, such as pollen exine formation, sporopollenin biosynthesis, pollen spermidine formation, and pollen tube formation/growth. A number of genes, including *homeobox gene 1* (*ATH1*), F box genes, phytohormone-related genes, and genes encoding TFs, with female-biased expression and implicated in female gametophyte development were also identified.

### KEGG pathway analysis in asparagus flower buds

**Fig. 5:**
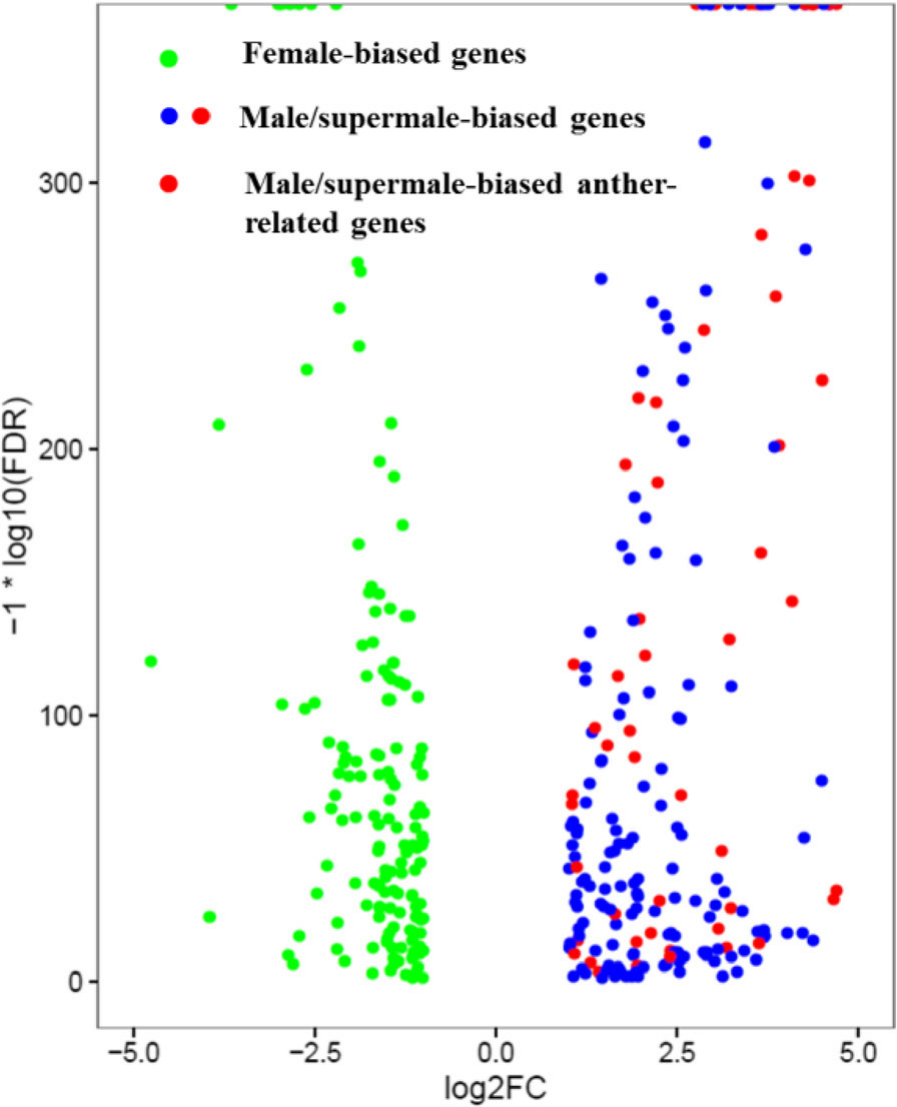
Volcano plot for differentially expressed genes involved in flower development between male/supermale and female flower buds. X axis represents the log2 (fold change value), while Y axis represents −1*log10 (FDR). Green dots show the female-biased flower-related genes, while blue and red dots represent the male/supermale-biased genes involved in flower development, among which, red dots represent anther-related male/supermale-biased genes

**Fig. 6:**
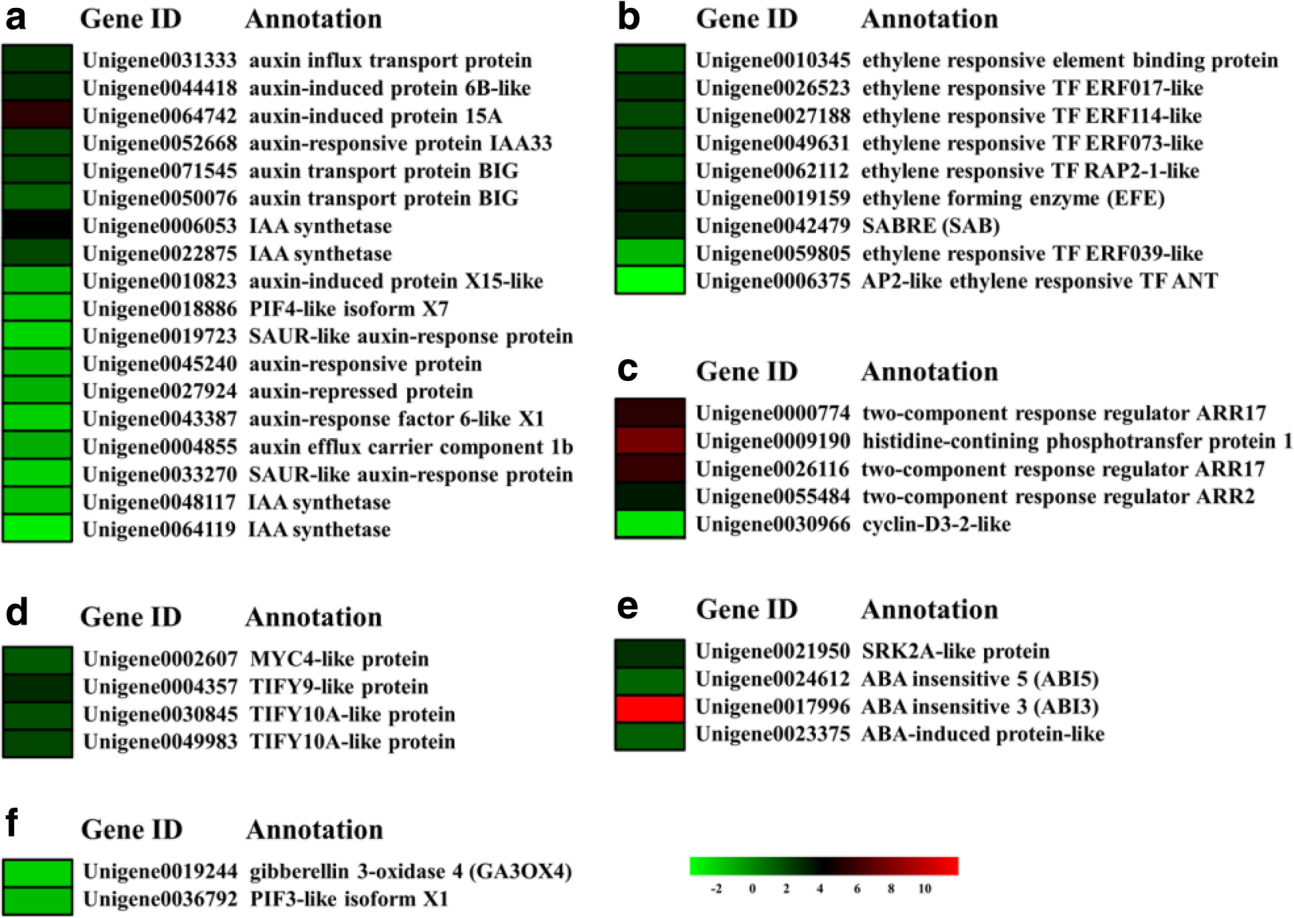
Heat map diagram of expression patterns for differentially expressed genes involved in several phytohormone signaling pathways, including auxin (a), ethylene (b), cytokinin (c), JA (d), ABA (e), and GA (f). Red and green colors indicate male/supermale-biased and female-biased genes, respectively

The KEGG analysis of the DEGs between male/supermale and female flower buds revealed that 300 unigenes were assigned to the top 18 KEGG biochemical pathways, ranked on the basis of *p*-value (Fig. 5). These pathways could be grouped into five classes, namely, “amino acid metabolism”, “biosynthesis of other secondary metabolites”, “carbohydrate metabolism”, “energy metabolism”, and “lipid metabolism”. A number of genes in the phenylpropanoid and flavonoid biosynthesis pathways were differentially expressed between male/supermale and female flower buds, and this observation is consistent with previous findings in spear tips [6]. Pathway-based analysis further showed that 43 DEGs were identified and implicated in “plant hormone signal transduction” pathway. Of these DEGs, 28 showed male/supermale-biased expression and 15 exhibited female-biased expression. These genes, including *ARR*, *ANT*, *SAUR*, and *ERF*, participated in the regulation of several hormone homeostasis and reproductive processes. Enrichment analysis demonstrated that most of these DEGs were involved in auxin, ethylene, cytokinin, and JA signaling pathways (Fig. 6, Additional file 8). In auxin signaling, 8 male/supermale-biased transcripts and 10 female-biased transcripts encoding auxin synthetases, auxin-induced proteins, auxin-responsive proteins, and auxin transport proteins were identified. Furthermore, 1 male/supermale-biased transcript encoding auxin influx transport protein and 2 female-biased transcripts encoding auxin-repressed protein and auxin efflux protein were detected. In ethylene signaling pathway, 3 male/supermale-biased unigenes and 1 female-biased unigene encoded an ethylene response transcription factor (ERF), and another female-biased unigene was an Ap2-like *ANT* gene. Of the 4 male/supermale-biased transcripts in the cytokinin signaling pathway, 3 were *ARR* genes and 1 was a gene encoding a histidine-containing phosphotransfer protein. Furthermore, 1 unigene responsible for a cyclin-D3–2-like protein was downregulated in female flower buds. In jasmonic acid (JA) signaling pathway, 3 TIFY-like genes and 1 MYC4-like gene were upregulated in male/supermale flower buds. Some DEGs related to other phytohormone signaling pathways were also detected.

### Differential expression of TFs in male and female flower buds

**Fig. 7:**
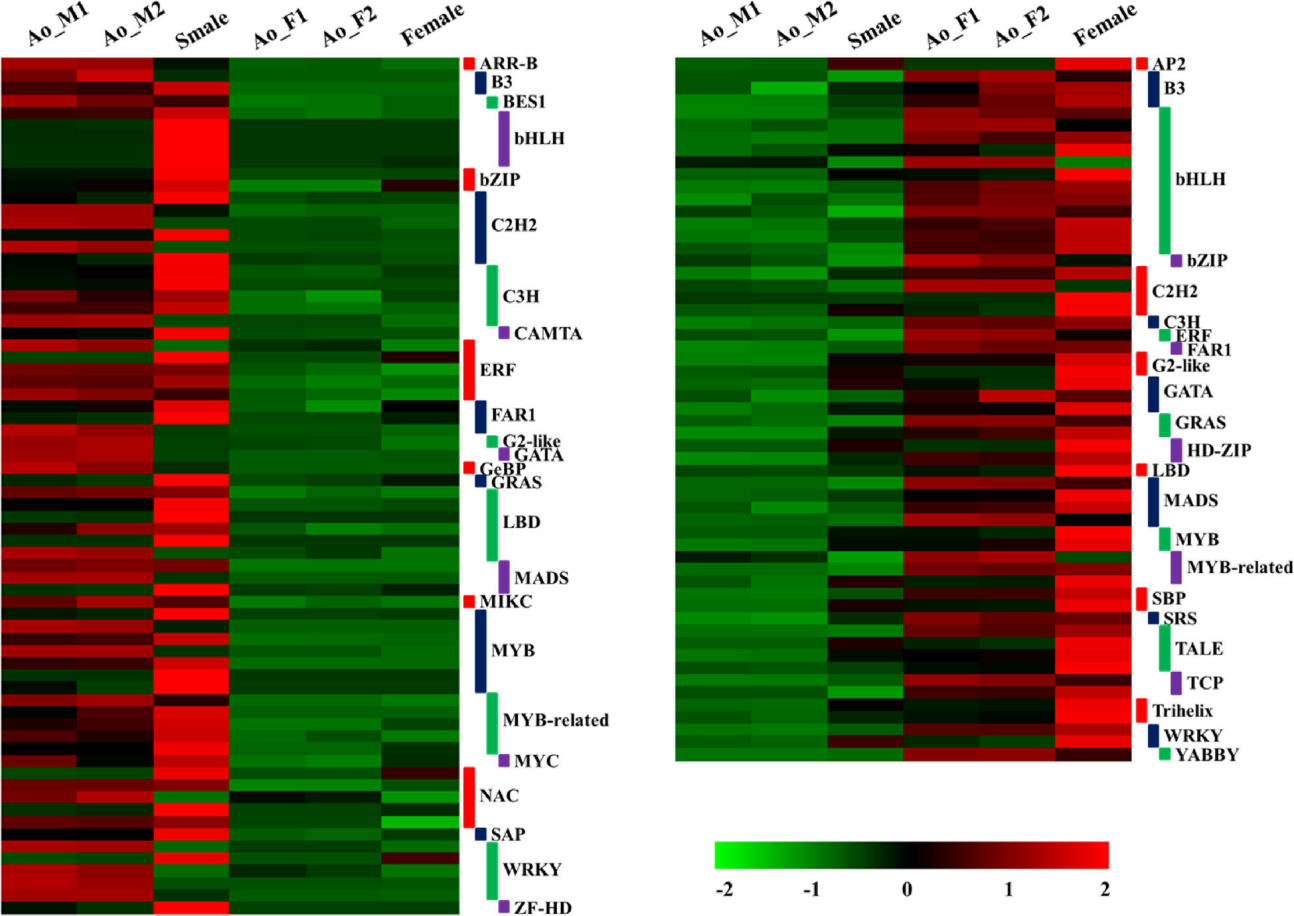
Differential expression of transcription factor gene between male/supermale and female flower buds. A variety of TF families showing differential expression (male/supermale-biased and female-biased in left and right panel, respectively) is shown on the heat map. The color scale represents the log-transformed RPKM value

A total of 128 TF genes were differentially expressed between male/supermale and female flower buds (Additional file 9). Of these genes, 70 were highly expressed in male/supermales and 58 were preferentially expressed in females. A heatmap showing the differential expression profiles of the TF genes between male/supermale and female flower buds is presented in Fig. 7. The differentially expressed TFs included members of C2H2, C3H, bZIP, ERF, bHLH, MADS, MYB, WRKY, NAC, and other small TF families. Among various families, bHLH and MYB families were the most predominant. Remarkably, 17 bHLH genes, including 5 with biased expression in male/supermales and 12 with biased expression in females, were differentially expressed between male/supermale and female flower buds. The MYB/MYB-related TF family members also exhibited a distinct differential expression pattern, that is, 12 were highly expressed in male/supermales and 5 were preferentially expressed in females. Members of other TF families, including C2H2, LBD, C3H, DRF, NAC, and WRKY, were also characterized by male/supermale-biased expression. Among the female-biased genes, C2H2 family genes were the most represented, followed by MADS and TALE family genes.

### miRNA-mRNA correlation analysis

Two small RNA libraries were constructed from a mixture of male and female flower buds. A total of 6,837,349 and 6,571,668 raw reads were generated from the male and female flower buds, respectively. After the adapter was trimmed, low-quality sequences were removed, and rRNAs, tRNAs, anRNA, and snoRNAs were filtered, the 2,949,390 and 2,902,544 remaining clean reads, representing 1,832,639 and 1,777,464 unique reads, were used to identify miRNA from male and female flower buds, respectively. Among these sequences, 72 known conserved miRNAs belonging to 53 families and 81 novel miRNAs were identified in the two samples (Additional file 10).

Differentially expressed miRNA (DEM) analysis revealed 15 upregulated DEMs (10 known and 5 novel) in male flower buds and 32 upregulated DEMs (12 known and 20 novel) in female flower buds. The DEM-DEG target pairs were detected from the transcriptome data based on the negative regulation between miRNA and their targets by combining DEM analysis results with RNA-seq data. A total of 90 targets showed opposite expression patterns with respect to 30 corresponding miRNAs (Additional file 10). In the male flower buds, 12 were upregulated DEMs with 57 downregulated target DEGs, including unigene0002102 (squamosa promoter-binding-like protein) and unigene0031841 (scarecrow-like protein), which are involved in flower development. In the female flower buds, 18 upregulated DEMs with 33 downregulated target DEGs, such as unigene0034398 (MADS-box transcription factor AP3) and unigene0001585 (transcription factor GAMYB-like protein), were identified.

## Discussion

In this study, flower buds were analyzed at the transcriptome level to enhance our understanding of the mechanisms of sex determination and differentiation in asparagus. Hence, we sequenced the cDNA collected from male and female flower buds, which might represent a critical stage of sex determination and differentiation in asparagus. Asparagus contains two types of male individuals: common XY type male and YY type supermale. These two types undergo a similar male flower development process. We hypothesized that the major factors that regulate sex determination and differentiation in males and supermales are identical or similar. Thus, the data obtained in our sequencing project were combined with previously reported findings on female and supermale flower buds in the pre-meiotic stage to identify DEGs between male/supermale and female flower buds. This combination and comparison could address the limitation of using only two replicates in our sequencing project. RT-qPCR validated that the filtered DEGs based on the comparison were accurate and reliable in different individuals and under various experimental conditions. These male/supermale-biased and female-biased genes are necessary to elucidate the underlying molecular variations regulating sex determination and differentiation.

### Male/supermale versus female flower bud expression patterns

We observed a number of genes that were overexpressed or specifically expressed either in male/supermale flower buds or female flower buds. The functional annotation of these DEGs revealed the molecular mechanisms of various flower-development-related biological processes, such as maintenance of floral organ identity, development of androecium and gynoecium, and development of gametophytes.

On the basis of the FlowerNet database, we annotated 284 homologs as flower-related genes. Of these homologs, 102 were identified as anther-related genes or male-sterile mutant-related genes. Several anther-related genes, such as *galacturonase* (*PG*) and *AMS*, were identified in accordance with a previous research [6]. This finding indicated that these genes were essential for anther development in different flower developmental stages. Anther-related cluster analysis further showed that 11 genes were associated with pollen wall formation. *Cation/H+ exchanger 19* (*CHX19*), *DUF064*, *SULTR*, *SYTB*, and *TSPO* were upregulated in pollen mother cell meiosis and involved in pollen exine formation [27]. *ACYL-COENZYME A SYNTHETASE* (*ACOS5*) and *CYTOCHROME P450* (*CYP704B1*) were implicated in sporopollenin biosynthesis. Knocked out *ACOS5* and *CYP704B1* do not produce pollens because of compromised pollen walls or the presence of irregular exine layers [28, 29]. *CYP86C3* and *Anther 7* (*A7*) were stamen specific in *Arabidopsis* and likely responsible for pollen spermidine formation. Our results further showed two stamen-specific genes, namely, *Arabidopsis LAT59 ORTHOLOG* (*AT59*) and *SUGAR TRANSPORTAER 11* (*STP11*), which played a role in pollen tube formation and growth [27]. Overall, these asparagus homologs showing male/supermale-biased expression might participate in pollen formation and development. However, genes responsible for pollen development are involved in downstream pathways and should not be implicated in gender determination.

A total of 149 homologs of flower development in *Arabidopsis* were significantly expressed to a greater extent in females than in males or supermales. These genes were implicated in gynoecium and female gametophyte development. Among the unigenes with differential expression between different sexual types, a large number of unknown genes did not yield a significant BLAST hit. These genes might include those specific to asparagus, those with unclear function in other plants, those with a large sequence diversity with other known genes, and other genes. With the elucidation of the functions of numerous genes, genes possibly involved in sex determination or differentiation in asparagus have been gradually understood. Hence, DEGs associated with flower development should be further examined.

### Roles of plant hormone signaling in sex differentiation in asparagus

Plant hormones are endogenous regulators with multiple signal functions that affect nearly all aspects of plant growth and development [30]. Various phytohormones are likely implicated in the regulation of sex-determining genes and developmental pathways in unisexual flowers [11]. In monoecious plants, such as cucumber (*Cucumis sativus*) and melon (*Cucumis melo*), unisexual flower development can be regulated by plant hormones that influence gender phenotypes. For example, ethylene represses male flower development [31], whereas GA promotes stamen development in female flowers [32]. In cucumber and melon, the classically identified *M* (masculinizing) and *F* (feminizing) loci are factors that modulate hormone signaling and promote organ abortion processes [33, 34]. Sexual development in dioecious species may be controlled by genetic variation and may be weakly sensitive to plant hormones. However, hormones may stimulate sexual differentiation in dioecious plants that have evolved from monoecious species [11]. In hemp (*Cannabis sativa*), the application of several plant hormones can influence their sexual phenotypes. For instance, GA induces masculinization, while IAA, ethylene, and kinetin elicit a feminization effect on the sex of hemp [35]. Thus, hormonal regulatory pathways should be examined to obtain useful information for the identification of sex-differentiating and sex-determining genes. In our study, the signaling pathways of several hormones, including auxin, ethylene, cytokinin, and JA, were enriched by pathway-based analysis (Fig. 5). The auxin pathway could interact with other pathways and play important roles in flower development [36]. A number of genes related to auxin metabolism and signaling were involved in auxin-mediated regulatory process of flowering [36]. The unique transcripts annotated as auxin-induced proteins, auxin-responsible proteins, and auxin-transport proteins were identified and differentially regulated between male/supermale and female flower buds of asparagus. Our results demonstrated that one auxin influx transport gene was expressed preferentially in male/supermale flower buds, whereas one auxin-repressed protein coding gene and one auxin efflux protein coding gene were more represented in female-biased flower buds than in male/supermale flower buds. These findings implied that auxin levels might be higher in male/supermale flower buds than in female asparagus flower buds, and auxin might play more important roles in the former than in the latter. This observation is in contrast to previous results demonstrating that auxins increase the percentage of female flowers in monoecious plants [37]. However, a floral organ that produces high concentrations of free auxin inhibits or retards the development of neighboring organs [38]. We predicted that high auxin levels in male/supermale flower buds inhibited the development of neighboring organs, such as gynoecium, and contributed to unisexual flower formation. Indeed, the effects and possible roles of auxin in plant sex regulation remarkably vary in different studies [39–41]. As such, the functions of auxin and its influence on sexual development should be further investigated. Other genes, such as *ERF*, *ARR*, and *TIFY*, were also found to be related to ethylene, cytokinin, and JA signaling. Overall, these results suggested that plant hormone-mediated transcriptional regulation may be essential for the sexual differentiation of asparagus. The characterization and future analysis of critical genes responsible for plant hormone production and signaling would greatly facilitate studies on the complex genetic network of sexual differentiation in asparagus.

### TF genes involved in sex determination and differentiation in asparagus

TFs play essential roles in the reproductive development of plants. Many genes, such as *AP1*, *AP2*, *ARF*, and *APL/NZZ*, which are involved in reproductive development, encode a TF [10, 42]. Indeed, *SRY*, a well-known sex-determining factor in human, encodes a TF [43]. Several sex determination- and sex differentiation-related genes in animals also encode TFs [44, 45]. Most importantly, the only identified and confirmed sex-determining gene encodes an HP-Zip type TF [5]. Growing studies suggested the essential roles of TFs in sex determination and differentiation, both in plants and animals. In our study, numerous TF genes represented significant differential expression between male/supermale and female asparagus flower buds. Among various TF families, zinc finger, MYB, NAC, bZIP, and bHLH were significantly enriched, and this enrichment indicated their crucial roles in sex determination or sex differentiation in asparagus. The members of these families have been demonstrated to be involved in sex differentiation in other dioecious or monoecious plants [5, 33]. In persimmons, the homeodomain TF gene *MeGI* belonging to HD-Zip family regulates anther fertility in a dosage-dependent pattern. *OGI* is a male-specific gene encoding a small RNA that targets *MeGI*, and the interplay between *OGI* and *MeGI* controls the gender of persimmons [5]. In our study, two HD-ZIP family TF genes (Unigene0023095 and Unigene0041406) were upregulated in female flower buds. A previous study showed that HD-Zip transcripts do not exhibit sex-biased expression in male and female asparagus spear tips [6], and these observations imply that these two HD-Zip genes may initiate biased transcription in flower buds other than in flower primordium. Although dioecy has evolved independently several times in different flowering plant lineages, further expression, genetic, and functional studies on these two TF genes should be performed to provide useful information for the understanding of sex determination or differentiation in asparagus.

The zinc finger TF family is composed of a large number of proteins containing one or more zinc finger domains and further classified into distinct subfamilies, such as C2H2 type, C3H type, and C2HC type [46, 47]. Proteins belonging to the zinc finger family participate in various regulatory processes [48, 49]. In melon, *CmWIP1*, which encodes a C2H2 zinc finger TF, promotes stamen development through an indirect repression of ethylene-stimulated *CmACS-7* gene. The interplay between *CmWIP1* and *CmACS-7* controls the development of male, female, and hermaphrodite flowers in melon [33]. In our study, six C2H2-type and five C3H-type TF genes showed male-biased expression, whereas five C2H2-type and one C3H type TF genes exhibited female-biased expression patterns. Remarkably, a clear *SUPERMAN-LIKE* homolog was four times more expressed in female flower buds than in male flower buds. In *Arabidopsis*, the *SUPERMAN* gene plays important roles in floral whorl and ovule development because of its negative activity during cell proliferation [50], and an increased number of stamens and infertile ovules are observed in *sup* mutants [51]. In the model dioecious plant *Silene latifolia*, the *SUPERMAN* homolog *SlSUP* is exclusively expressed in female flower and implicated in female flower development [52].

bHLH family members play diverse roles in vegetative growth, reproductive growth, plant growth, morphogenesis, and stress responses [53, 54]. Among the differentially expressed bHLH genes in male and female asparagus flower buds, five showed predominant expression in males. One of these genes was the ortholog of *AMS*, a gene required for male reproductive development, expressed specifically in the tapetum of *Arabidopsis* anthers, and involved in tapetal tissue development [55]. *AMS* can also regulate the expression of several other anther-development-related genes, such as *bHLH89* and *bHLH91* [55]. We also found that *bHLH89* was preferentially expressed in males or supermales. Hence, we proposed that the interaction of *bHLH89* and *AMS* was implicated in anther development in asparagus. Furthermore, 11 bHLH genes, including orthologs of PHYTOCHROME INTERACTING FACTOR4 (*PIF4*), *GLABRA3*, *bHLH57*, *bHLH66*, *bHLH74*, and *bHLH75*, which participate in flowering time regulation, hormonal regulation, stress response, and cell differentiation, were significantly and highly expressed in female flower buds [56–58].

The MYB/MYB-related family of TF genes is implicated in various important plant biological processes, including flower development [59]. Twelve and five MYB/MYB-related transcripts were preferentially expressed in male/supermale and female flower buds, respectively. Among these transcripts, two are homologs of *SWI/SNF* that encodes the proteins of chromatin remodeling complexes essential for leaf and flower development. This kind of TF could regulate the expression pattern of numerous floral organ identity genes, such as *APETALA1*, *APETALA3*, and *AGL24* [60]. Recently, Murase et al. briefly reported a MYB TF gene *MSE1* which may be involved in sex determination in asparagus. *MSE1* is specifically expressed in male asparagus. Knockout of the *MSE1* homolog in *Arabidopsis* causes male sterility [19]. However, this is only a preliminary study, validation of the function of this gene and investigation of the underlying mechanism are in need of further research.

A number of TF genes belonging to other families, such as MADS, WRKY, GATA, NAC, LBD, AP, and SAP, also exhibit a biased expression in male/supermale or female flower buds. Therefore, these genes also function in sex determination or differentiation in asparagus. For example, an APETALA2 (AP2)-like ethylene-responsive TF gene *AINTEGUMENTA* (*ANT*) is preferentially expressed in females compared with male or supermales. *ANT* participates in many aspects of female development, including ovule and gynoecium development [61, 62]. There were also a number of differentially expressed TFs with unclear functions in other plants. As such, these TFs are considered as potential candidates that are possibly involved in male and female flower development. Further genetic and functional these genes will provide relevant information to elucidate the sex determination and differentiation in this dioecious species.

### miRNAs involved in sexual regulation in asparagus

miRNAs are key gene expression regulators that play important roles in multiple cellular processes. miRNAs are likely involved in animal reproduction and sexual differentiation [63, 64] and plant development processes, including flower development [65–67]. Recently, Chen et al. identified a number of miRNAs and their targets, which may be involved in reproductive organ development in asparagus through small RNA sequencing and degradome analysis [21]. In this study, small RNAs were sequenced, miRNAs were identified, and correlation analysis between miRNA and transcriptome was performed to identify the potential roles of miRNAs in regulating sex differentiation in asparagus.

Various DEMs, including miR156, miR159, miR165, miR172, and miR319, which have been reported to be involved in flower development [68, 69], were identified between male and female flower buds. The targets of the DEMs were predicted, and a number of DEG-DEM pairs, such as miR159-GAMYB-like protein, miR156-squamosa promoter-binding-like protein, miR171-scarecrow-like protein, and miR5719-MADS-box transcription factor AP3, were identified.

*Arabidopsis GAMYB*-like genes, such as *MYB33* and *MYB65*, regulated by miR159, are essential for anther development [70]. Mutant analysis demonstrated that the anther development of *myb33myb65* double mutant is blocked in the premeiotic stage and is accounted for male sterility. Although the overexpression of miR159a in *Arabidopsis* also causes male sterility, they induce the formation of anther phenotypes that differ from those of *myb33 myb65*. These findings indicated that other members of the *Arabidopsis GAMYB*-like family are targeted by miR159 and implicated in anther development [70]. A number of *GAMYB*-like transcripts were identified in this study (Additional file 9). Consistent with previous findings, our results showed that patterns of the *GAMYB*-like transcripts were upregulated and miR159 was downregulated in male flower buds. These findings raise the possibility that miRNA-regulated *GAMYB*-like genes were involved in anther development in male asparagus, and the upregulated miRNA and downregulated *GAMYB*-like genes might participate in the androecium abortion of the female flower buds of asparagus. Several unknown DEGs were also targeted by DEMs. Hence, these genes might be involved in sex determination and differentiation, but this possibility has yet to be verified.

## Conclusions

RNA-Seq technology was applied to systematically investigate the sex-biased gene expression in the reproductive organs of dioecious asparagus. Combined with the data of a previous study, our results revealed 4876 DEGs between male/supermale and female flower buds. A number of candidate genes related to plant reproduction, plant hormone signaling, and TFs were characterized and implicated in complex networks of sex determination and differentiation. Our transcriptome analysis provided new insights into the genetic regulation of asparagus sex expression. Our study also established a basis for further genomic research on this important dioecious vegetable crop.

## Methods

### Plant materials

The male and female asparagus (variety ‘UC309’) used in this study were grown in a greenhouse at Henan Normal University. Six male and six female asparagus individuals from a full-sibling progeny were sampled to represent two replicates of two sex types. Flower buds (0.5–0.7 mm in diameter), which may represent a critical sex determination stage from each sex type [21, 71], were collected, immediately frozen in liquid nitrogen, and stored at −80 °C until use.

### Illumina sequencing and data processing

Total RNA was extracted from asparagus flower buds by using TRIZOL reagent (Life Technologies, CA, USA) in accordance with the manufacturer’s instructions. In each repetition, RNA was isolated from a mixture of flower buds from three individual plants in equal quantity. Approximately 3 μg of RNA per sample was used to construct the sequencing library with a NEBNext® Ultra™ Directional RNA Library Prep Kit for Illumina® (NEB, USA) in accordance with the manufacturer’s recommendations. Transcriptome sequencing was performed on an Illumina Hiseq 2500 platform, and 125 bp paired-end reads were generated.

Raw reads in a fastq format, including our sequencing data and previously reported data on flower buds [6], were processed through in-house perl scripts. In this step, clean data (clean reads) were obtained by removing reads containing an adapter, reads comprising at least 10 Ns, and low-quality reads from raw data. Downstream analyses were based on high-quality clean data. De novo assembly was conducted using Trinity with min_kmer_cov set to 2 by default, and all of the other parameters were set in default [72]. To evaluate the transcriptome completeness, BUSCO v3 [73] was used by using the ortholog database for plants, which includes 1440 genes. We also used Transrate v1.0.3 [74] to assess the transcriptome assembly quality.

### Gene annotation and function classification

Gene function was annotated on the basis of the following five databases by using BLAST with a cutoff E-value of 10^−5^: Nr (NCBI non-redundant protein sequences), KOG/COG (Clusters of Orthologous Groups of Proteins), Swiss-Prot (a manually annotated and reviewed protein sequence database), KEGG Ortholog database, and GO.

### Analysis of flower-related genes based on *Arabidopsis* data

A dataset comprising a large number of flower-related genes of *Arabidopsis* was generated to investigate the flower-related genes in the assembled unigenes of asparagus flower buds. These data were obtained mainly from a plant reproduction database [75] and a flower-related database [27]. After the redundant genes were removed, 11,398 non-redundant *Arabidopsis* gene loci were involved in the dataset. The *Arabidopsis* proteome was downloaded from The Arabidopsis Information Resource (TAIR) and set as a reference for local BLASTX analysis performed by using the asparagus assembled unigenes as the query.

### Differential expression analysis

Sequencing reads were mapped to the assembled sequences by using SOAPaligner/soap2 [76]. Read counts were normalized by calculating the RPKM value of each unigene in different samples. Differential expression analysis was performed using the DESeq R package (1.10.1) [77]. *p*-value was adjusted to the false discovery rate (FDR) for multiple testing [78]. Unigenes with a minimal twofold difference in expression (|log_2_ Ratio| ≥ 1) and FDR of ≤0.05 were considered as DEGs. The intersection of our data and Harkess’s data for pre-meiotic flower buds was identified as female- and male/supermale- biased unigenes, which were used for further analysis. These DEGs were then subjected to GO enrichment analysis in GO seq R packages based on Wallenius non-central hyper-geometric distribution [79], which can be adjusted for gene length bias in DEGs. The statistical enrichment of DEGs in KEGG pathways was evaluated using KOBAS software [80]. The assembled unigenes were analyzed with Hidden Markov Model (HMM) profiles based on plant TF database (PlantTFDB) by using HMMER v3.1b2 (http://hmmer.org/) to identify differentially expressed TFs between male and female flower buds. Potential sex-specific or differentially expressed TFs were detected and further examined.

### qRT-PCR verification

Twenty-four DEGs were selected for quantitative RT-PCR analysis by using the RNA samples of flower buds from individuals different from those used for transcriptome sequencing to validate our Illumina sequencing data. Gene-specific primers were designed according to the reference unigene sequences by using Primer 6 (Additional file 11). 18S rDNA gene was used as an internal control for qPCR analysis. cDNA synthesize and q-PCR were performed using a previously described procedure [81]. The expression levels were determined with 2^−ΔΔCT^ method [82].

### Small RNA sequencing and miRNA identification

Two independent small RNA libraries from asparagus male and female flower buds were constructed and sequenced. For each library, three male individual plants or three female individual plants in equal quantity were pooled. Total RNA (3 μg) was used to construct a sRNA library for each male and female sample by using NEBNext Multiplex Small RNA Library Prep Set for Illumina (NEB, USA). The prepared libraries were sequenced on an Illumina Hiseq 2500 platform and 50 bp single-end reads were generated. The sequencing data, including transcriptome sequencing and sRNA sequencing results, were deposited into the NCBI SRA database with accession numbers of SRR5112683, SRR5112684, SRR5118382, and SRR5118383.

### Identification and differential expression analysis of known and novel miRNAs

Raw reads were processed and filtered by using Illumina’s Genome Analyzer Pipeline V1.5. The filtered small RNA sequences were mapped to asparagus mRNAs, Rfam (http://rfam.xfam.org/), and Repbase (http://www.girinst.org/repbase/) to discard mRNA, rRNA, tRNA, snRNA, snoRNA, and repeat sequences. Known miRNAs and novel miRNAs were identified using the methods described by a previous research [83].

The miRNAs in male and female flower buds were subjected to differential expression analysis by using the DEGseq [84] R package. *p*-value was adjusted using a *q*-value [85]. *q*-value of <0.01 and |log_2_(foldchange)| > 1 were set as the threshold for significantly differential expression analysis by default.

### miRNA target prediction

A plant miRNA target prediction server (http://plantgrn.noble.org/psRNATarget/) was used to predict putative miRNA target genes with default parameters based on the assembled data of the asparagus transcriptome.

## Additional files

Additional file 1: Figure S1. Overview of length distribution of assembled unigenes in asparagus flower buds. (TIFF 1080 kb)

Additional file 2: Figure S2. BUSCO assessment results of the transcriptome assembly. (TIFF 58 kb)

Additional file 3: Table S1. All unigenes assembled based on transcripotome sequencing data of asparagus flower buds. (XLSX 13949 kb)

Additional file 4: Table S2. Male/supermale-biased DEG unigenes in asparagus flower buds. **Table S3** Female-biased DEG unigenes in asparagus flower buds. (XLSX 1016 kb)

Additional file 5: Table S4. Male/supermale-specific expressed unigenes in asparagus flower buds. **Table S5.** Female-specific unigenes in asparagus flower buds. (XLSX 43 kb)

Additional file 6: Figure S3. Functional classification of DEGs in male/supermale and female flower buds. (TIFF 755 kb)

Additional file 7: Table S6. Male/supermale-biased unigenes which were annotated as flower-related genes. **Table S7** Female-biased unigenes which were annotated as flower-related genes. (XLSX 44 kb)

Additional file 8: Table S8. Differentially expressed phytohormone-related unigenes between male/supermale and female asparagus flower buds. (XLSX 13 kb)

Additional file 9: Table S9. Differentially expressed transcription factor unigenes between male/supermale and female asparagus flower buds. (XLSX 26 kb)

Additional file 10: Table S10. Identified known and novel miRNAs in asparagus flower buds. **Table S11.** miRNA-mRNA correlation analysis in asparagus flower buds. (XLSX 24 kb)

Additional file 11: Table S12. Primers for q-PCR. (XLSX 16 kb)

## Abbreviations

BLAST: Basic local alignment search tool
COG: Clusters of orthologous groups of proteins
DEGs: Differentially expressed genes
DEM: Differentially expressed miRNA
ERF: Ethylene response transcription factor
FDR: False discovery rate
GO: Gene ontology
HMM: Hidden markov model
JA: Jasmonic acid
KEGG: Kyoto encyclopedia of genes and genomes
miRNA: microRNA
Nr: NCBI non-redundant protein sequences
PCR: Polymerase chain reaction
RPKM: The read per kilobase per million
SEGs: Sex-specific expressed genes
TAIR: The Arabidopsis information resource
TF: Transcription factor

## References

[1] Renner SS (2014). The relative and absolute frequencies of angiosperm sexual systems: dioecy, monoecy, gynodioecy, and an updated online database. Am J Bot.

[2] Ming R, Wang J, Moore PH, Paterson AH (2007). Sex chromosomes in flowering plants. Am J Bot.

[3] Li SF, Zhang GJ, Yuan JH, Deng CL, Gao WJ (2016). Repetitive sequences and epigenetic modification: inseparable partners play important roles in the evolution of plant sex chromosomes. Planta.

[4] Charlesworth D (2013). Plant sex chromosome evolution. J Exp Bot.

[5] Akagi T, Henry IM, Comai L (2014). A Y-chromosome-encoded small RNA acts as a sex determinant in persimmons. Science.

[6] Harkess A, Mercati F, Shan HY, Sunseri F, Falavigna A, Leebens-Mack J (2015). Sex-biased gene expression in dioecious garden asparagus (*Asparagus officinalis*). New Phytol.

[7] Poethig RS, Jr Coe EH, Johri MM (1986). Cell lineage patterns in maize embryogenesis: a clonal analysis. Dev Biol.

[8] Walbot V, Evans MMS (2003). Unique features of the plant life cycle and their consequences. Nat Rev Genet.

[9] Chawla A, Stobdan T, Srivastava RB, Jaiswal V, Chauhan RS, Kant A (2015). Sex-biased temporal gene expression in male and female floral buds of seabuckthorn (*Hippophae rhamnoides*). PLoS One.

[10] Rocheta M, Sobral R, Magalhães J, Amorim MI, Ribeiro T, Pinheiro M, Egas C, Morais-Cecílio L, Costa MMR (2014). Comparative transcriptomic analysis of male and female flowers of monoecious *Quercus suber*. Front Plant Sci.

[11] Diggle PK, Di Stilio VS, Gschwend AR, Golenberg EM, Moore RC, Russell JRW, Sinclair JP (2011). Multiple developmental processes underlie sex differentiation in angiosperms. Trends Genet.

[12] Sekido R, Lovell-Badge R (2008). Sex determination involves synergistic action of SRY and SF1 on a specific *Sox9* enhancer. Nature.

[13] Arumuganathan K, Earle ED (1991). Nuclear DNA content of some important plant species. Plant Mol Biol Rep.

[14] Rick CM, Hanna GC (1943). Determination of sex in *Asparagus officinalis* L. Am J Bot.

[15] Flory WS (1932). Genetic and cytological investigations on *Asparagus officinalis* L. Genet Princeton.

[16] Jiang C, Lewis ME, Sink KC (1997). Combined RAPD and RFLP molecular linkage map of asparagus. Genome.

[17] Reamon-Büttner SM, Schondelmaier J, Jung C (1998). AFLP markers tightly linked to the sex locus in *Asparagus officinalis* L. Mol Breeding.

[18] Telgmann-Rauber A, Jamsari A, Kinney MS, Pires JC, Jung C (2007). Genetic and physical maps around the sex-determining *M*-locus of the dioecious plant asparagus. Mol Gen Genomics.

[19] Murase K, Shigenobu S, Fujii S, Ueda K, Murata T, Sakamoto A, Wada Y, Yamaguchi K, Osakabe Y, Osakabe K, Kanno A, Ozaki Y, Takayama S (2017). MYB transcription factor gene involved in sex determination in *Asparagus officinalis*. Genes Cells.

[20] Mercati F, Riccardi P, Leebens-Mack J, Abenavoli MR, Falavigna A, Sunseri F (2013). Single nucleotide polymorphism isolated from a novel EST dataset in garden asparagus (*Asparagus officinalis* L.). Plant Sci.

[21] Chen J, Zheng Y, Qin L, Wang Y, Chen L, He Y, Fei Z, Lu G (2016). Identification of miRNAs and their targets through high-throughput sequencing and degradome analysis in male and female *Asparagus officinalis*. BMC Plant Biol.

[22] Yan H, Zhang H, Wang Q, Jian H, Qiu X, Baudino S, Just J, Raymond O, Gu L, Wang J, Bendahmane M, Tang K (2016). The *Rosa chinensis* cv. Viridiflora phyllody phenotype is associated with misexpression of flower organ identity genes. Front Plant Sci.

[23] Shang C, Bi G, Yuan Z, Wang Z, Alam MA, Xie J (2016). Discovery of genes for production of biofuels through transcriptome sequencing of *Dunaliella parva*. Algal Res.

[24] Liu J, Yin T, Ye N, Chen Y, Yin T, Liu M, Hassani D (2013). Transcriptome analysis of the differentially expressed genes in the male and female shrub willows (*Salix suchowensis*). PLoS One.

[25] Urasaki N, Tarora K, Shudo A, Ueno H, Tamaki M, Miyagi N, Adaniya S, Matsumura H (2012). Digital transcriptome analysis of putative sex-determination genes in papaya (*Carica papaya*). PLoS One.

[26] Lu J, Luan P, Zhang X, Xue S, Peng L, Mahbooband S, Sun X (2014). Gonadal transcriptomic analysis of yellow catfish (*Pelteobagrus fulvidraco*): identification of sex-related genes and genetic markers. Physiol Genomics.

[27] Pearce S, Ferguson A, King J, Wilson ZA (2015). FlowerNet: a gene expression correlation network for anther and pollen development. Plant Physiol.

[28] de Azevedo SC, Kim SS, Koch S, Kienow L, Schneider K, McKim SM, Haughn GW, Kombrink E, Douglas CJ (2009). A novel fatty acyl-CoA synthetase is required for pollen development and sporopollenin biosynthesis in *Arabidopsis*. Plant Cell.

[29] Dobritsa AA, Shrestha J, Morant M, Pinot F, Matsuno M, Swanson R, Moller BL, Preuss D (2009). CYP704B1 is a long-chain fatty acid ω-hydroxylase essential for sporopollenin synthesis in pollen of Arabidopsis. Plant Physiol.

[30] Gray WM (2004). Hormonal regulation of plant growth and development. PLoS Biol.

[31] Ando S, Sato Y, Kamachi S, Sakai S (2001). Isolation of a MADS-box gene (*ERAF*_*17*_) and correlation of its expression with the induction of formation of female flowers by ethylene in cucumber plants (*Cucumis sativus* L.). Planta.

[32] Peterson CE, Anhder LD (1960). Induction of staminate flowers on gynoecious cucumbers with gibberellin A3. Science.

[33] Martin A, Troadec C, Boualem A, Rajab M, Fernandez R, Morin H, Pitrat M, Dogimont C, Bendahmane A (2009). A transposon-induced epigenetic change leads to sex determination in melon. Nature.

[34] Mibus H, Tatlioglu T (2004). Molecular characterization and isolation of the F/f gene for femaleness in cucumber (*Cucumis sativus* L.). Theor Appl Genet.

[35] Galoch E (1978). The hormonal control of sex differentiation in dioecious plants of hemp (*Cannabis sativa*). The influence of plant growth regulators on sex expression in male and female plants. Acta Soc Bot Pol.

[36] Zhao Y (2010). Auxin biosynthesis and its role in plant development. Annu Rev Plant Biol.

[37] Galun E, Izhar S, Atsmon D (1965). Determination of relative auxin content in hermaphrodite and andromonocious *Cucumis sativus* L. Plant Physiol.

[38] Aloni R, Aloni E, Langhans M, Ullrich CI (2006). Role of auxin in regulating *Arabidopsis* flower development. Planta.

[39] Takasi S, Hideo I (1964). Factors responsible for the sex expression of the cucumber plant: 14. Auxin and gibberellin content in the stem apex and the sex pattern of flowers. Tohoku J Agu Res.

[40] Ćulafić L, Nešković M (1974). Indole auxins in spinach plants grown in long and short days. Biol Plant.

[41] Goroshkevich SN, Menyailo LN (1996). Phytohormonal gradients as a factor controlling the differentiation of cedar pine crown into generative stories. Fiziol Rast.

[42] Cheng X, Peng J, Ma J, Tang Y, Chen R, Mysore KS, Wen J (2012). *NO APICAL MERISTEM* (*MtNAM*) regulates floral organ identity and lateral organ separation in *Medicago truncatula*. New Phytol.

[43] Teo SH, Grasser KD, Thomas JO (1995). Differences in the DNA-binding properties of the hmg-box domains of HMG1 and the sex-determining factor SRY. FEBS J.

[44] Smith CA, Roeszler KN, Ohnesorg T (2009). The avian Z-linked gene *DMRT1* is required for male sex determination in the chicken. Nature.

[45] Chong T, Collins JJ, Brubacher JL, Zarkower D, Newmark PA (2013). A sex-specific transcription factor controls male identity in a simultaneous hermaphrodite. Nat Commun.

[46] Takatsuji H (1998). Zinc-finger transcription factors in plants. Cell Mol Life Sci.

[47] Ciftci-Yilmaz S, Mittler R (2008). The zinc finger networks of plants. Cell Mol Life Sci.

[48] Kobayashi A, Sakamoto A, Kubo K, Rybka Z, Kanno Y, Takatsuji H (1998). Seven zinc-finger transcription factors are expressed sequentially during the development of anthers in petunia. Plant J.

[49] Rizhsky L, Davletova S, Liang H, Mittler R (2004). The zinc finger protein Zat12 is required for cytosolic ascorbate peroxidase1 expression during oxidative stress in Arabidopsis. J Biol Chem.

[50] Gaiser JC, Robinson-Beers K, Gasser CS (1995). The Arabidopsis *SUPERMAN* gene mediates asymmetric growth of the outer integument of ovules. Plant Cell.

[51] Sakai H, Medrano LJ, Meyerowitz EM (1995). Role of *SUPERMAN* in maintaining Arabidopsis floral whorl boundaries. Nature.

[52] Kazama Y, Fujiwara MT, Koizumi A, Nishihara K, Nishiyama R, Kifune E, Abe T, Kawano S (2009). A *SUPERMAN*-like gene is exclusively expressed in female flowers of the dioecious plant *Silene latifolia*. Plant Cell Physiol.

[53] Heim MA, Jakoby M, Werber M, Martin C, Weisshaar B, Bailey PC (2003). The basic helix-loop-helix transcription factor family in plants: a genome-wide study of protein structure and functional diversity. Mol Biol Evol.

[54] Sharma N, Xin R, Kim DH, Sung S, Lange T, Huq E (2016). NO FLOWERING SHORT DAY (NFL) is a bHLH transcription factor that promotes flowering specifically under short-day conditions in *Arabidopsis*. Development.

[55] Xu J, Yang C, Yuan Z, Zhang D, Gondwe MY, Ding Z, Liang W, Zhang D, Wilson ZA (2010). The *ABORTED MICROSPORES* regulatory network is required for postmeiotic male reproductive development in *Arabidopsis thaliana*. Plant Cell.

[56] Proveniers MCG, van Zanten M (2013). High temperature acclimation through PIF4 signaling. Trends Plant Sci.

[57] Matías-Hernádez L, Aguilar-Jaramillo AE, Cigliano RA, Sanseverino W, Pelaz S (2016). Flowering and trichome development share hormonal and transcription factor regulation. J Exp Bot.

[58] Castilhos G, Lazzarotto F, Spagnolo-Fonini L, Bodanese-Zanettini MH, Margis-Pinheiro M (2015). Possible roles of basic helix-loop-helix transcription factors in adaptation to drought. Plant Sci.

[59] Vimolmangkang S, Han Y, Wei G, Korban SS (2013). An apple MYB transcription factor, *MdMYB*_*3*_, is involved in regulation of anthocyanin biosynthesis and flower development. BMC Plant Biol.

[60] Sacharowski SP, Gratkowska DM, Sarnowska EA, Kondrak P, Jancewica I, Porri A, Bucior E, Rolicka AT, Franzen R, Kowalczyk J, Pawlikowska K, Huettel B, Torti S, Schmelzer E, Coupland G, Jerzmanowski A, Koncz C, Sarnowski TJ (2015). Swp_73_ subunits of Arabidopsis SWI/SNF chromatin remodeling complexes play distinct roles in leaf and flower development. Plant Cell.

[61] Elliott R, Betzner A, Huttner E (1996). AINTEGUMENTA, an APETALA2-like gene of Arabidopsis with pleiotropic roles in ovule development and floral organ growth. Plant Cell.

[62] Liu Z, Franks RG, Klink VP (2000). Regulation of gynoecium marginal tissue formation by *LEUNIG* and *AINTEGUMENTA*. Plant Cell.

[63] Torley K, da Silveira JC, Smith P, Anthony RV, Veeramachaneni DNR, Winger QA, Bouma GJ (2011). Expression of miRNAs in ovine fetal gonads: potential role in gonadal differentiation. Reprod Biol Endocrin.

[64] Cutting AD, Bannister SC, Doran TJ, Sinclair AH, Tizard MVL, Smith CA (2012). The potential role of microRNAs in regulating gonadal sex differentiation in the chicken embryo. Chromosom Res.

[65] Spanudakis E, Jackson S (2014). The role of microRNAs in the control of flowering time. J Exp Bot.

[66] Chen X (2004). A microRNA as a translational repressor of *APETALA2* in *Arabidopsis* flower development. Science.

[67] Wollmann H, Mica E, Todesco M, Long JA, Weigel D (2010). On reconciling the interactions between *APETALA2*, miR172 and *AGAMOUS* with the ABC model of flower development. Development.

[68] Luo Y, Guo Z, Li L (2013). Evolutionary conservation of microRNA regulatory programs in plant flower development. Dev Biol.

[69] Nag A, King S, Jack T (2009). miR319a targeting of *TCP4* is critical for petal growth and development in *Arabidopsis*. P Natl Acad Sci USA.

[70] Millar AA, Gubler F (2005). The Arabidopsis *GAMYB-Like* genes, *MYB33* and *MYB65*, are microRNA-regulated genes that redundantly facilitate anther development. Plant Cell.

[71] Caporali E, Carboni A, Galli MG, Rossi G, Spada A, Marziani Longo GP (1994). Development of male and female flower in *Asparagus officinalis*: search for point of transition from hermaphroditic to unisexual developmental pathway. Sex Plant Reprod.

[72] Simão FA, Waterhouse RM, Ioannidis P, Kriventseva EV, Zdobnov EM (2015). BUSCO: assessing genome assembly and annotation completeness with single-copy orthologs. Bioinformatics.

[73] Smith-Unna R, Boursnell C, Patro R, Hibberd JM, Kelly S (2016). TransRate: reference-free quality assessment of de novo transcriptome assemblies. Genome Res.

[74] Grabherr MG, Haas BJ, Yassour M, Levin JZ, Thompson DA, Amit I, Adiconis X, Fan L, Raychowdhury R, Zeng Q, Chen Z, Mauceli E, Hacohen N, Gnirke A, Rhind N, di Palma F, Birren BW, Nusbaum C, Lindblad-Toh K, Friedman N, Regev A (2011). Full-length transcriptome assembly from RNA-Seq data without a reference genome. Nat Biotechnol.

[75] Galla G, Vogel H, Sharbel TF, Barcaccia G (2015). De novo sequencing of the Hypericum perforatum L flower transcriptome to identify potential genes that are related to plant reproductive sensu lato. BMC Genomics.

[76] Li R, Yu C, Li Y, Lam TW, Yiu SM, Kristiansen K, Wang J (2009). SOAP2: an improved ultrafast tool for short read alignment. Bioinformatics.

[77] Anders S, Huber W (2010). Differential expression analysis for sequence count data. Genome Biol.

[78] Benjamini Y, Hochberg Y (1995). Controlling the false discovery rate: a practical and powerful approach to multiple testing. J R StatistSoc B.

[79] Young MD, Wakefield MJ, Sayth GK, Oshlack A (2010). Method gene ontology analysis for RNA-seq: accounting for selection bias. Genome Biol.

[80] Mao X, Cai T, Olyarchuk JG, Wei L (2005). Automated genome annotation and pathway identification using the KEGG Orthology (KO) as a controlled vocabulary. Bioinformatics.

[81] Deng CL, Wang NN, Li SF, Dong TY, Zhao XP, Wang SJ, Gao WJ, Lu LD (2015). Isolation of differentially expressed sex genes in garden asparagus using suppression subtractive hybridization. J Plant Res.

[82] Livak KJ, Schmittgen TD (2001). Analysis of relative gene expression data using real-time quantitative PCR and the 2^-ΔΔCT^ method. Methods.

[83] Li H, Dong Y, Chang J, He J, Chen H, Liu Q, Wei C, Ma J, Zhang Y, Yang J, Zhang X (2016). High-throughput microRNA and mRNA sequencing reveals that microRNAs may be involved in melatonin-mediated cold tolerance in *Citrullus lanatus* L. Front Plant Sci.

[84] Wang L, Feng Z, Wang X, Wang X, Zhang X (2010). DEGseq: an R package for identifying differentially expressed genes from RNA-seq data. Bioinformatics.

[85] Storey JD (2003). The positive false discovery rate: a Bayesian interpretation and the q-value. Ann Stat.

