# Supplementary figures and images for: Comparative transcriptome analysis reveals differentially expressed genes associated with sex expression in garden asparagus (Asparagus officinalis)
Source: BMC Plant Biol. 2017 Aug 22;17:143. doi: 10.1186/s12870-017-1091-6 (PMC5567890; doi:10.1186/s12870-017-1091-6)

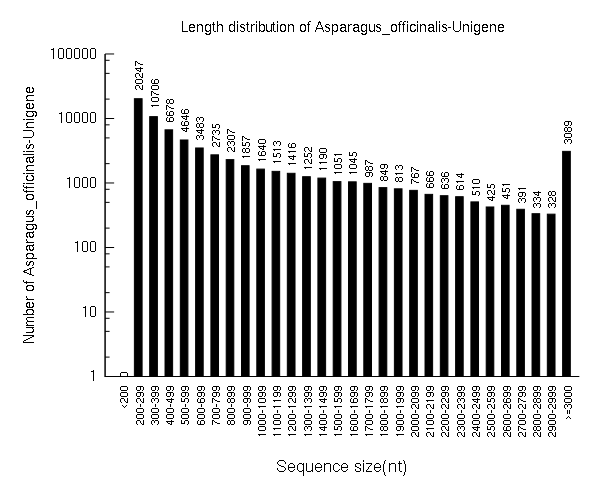

Supplement: Supplementary file 1 — Overview of length distribution of assembled unigenes in asparagus flower buds. (TIFF 1080 kb) [file 12870_2017_1091_MOESM1_ESM.tif]

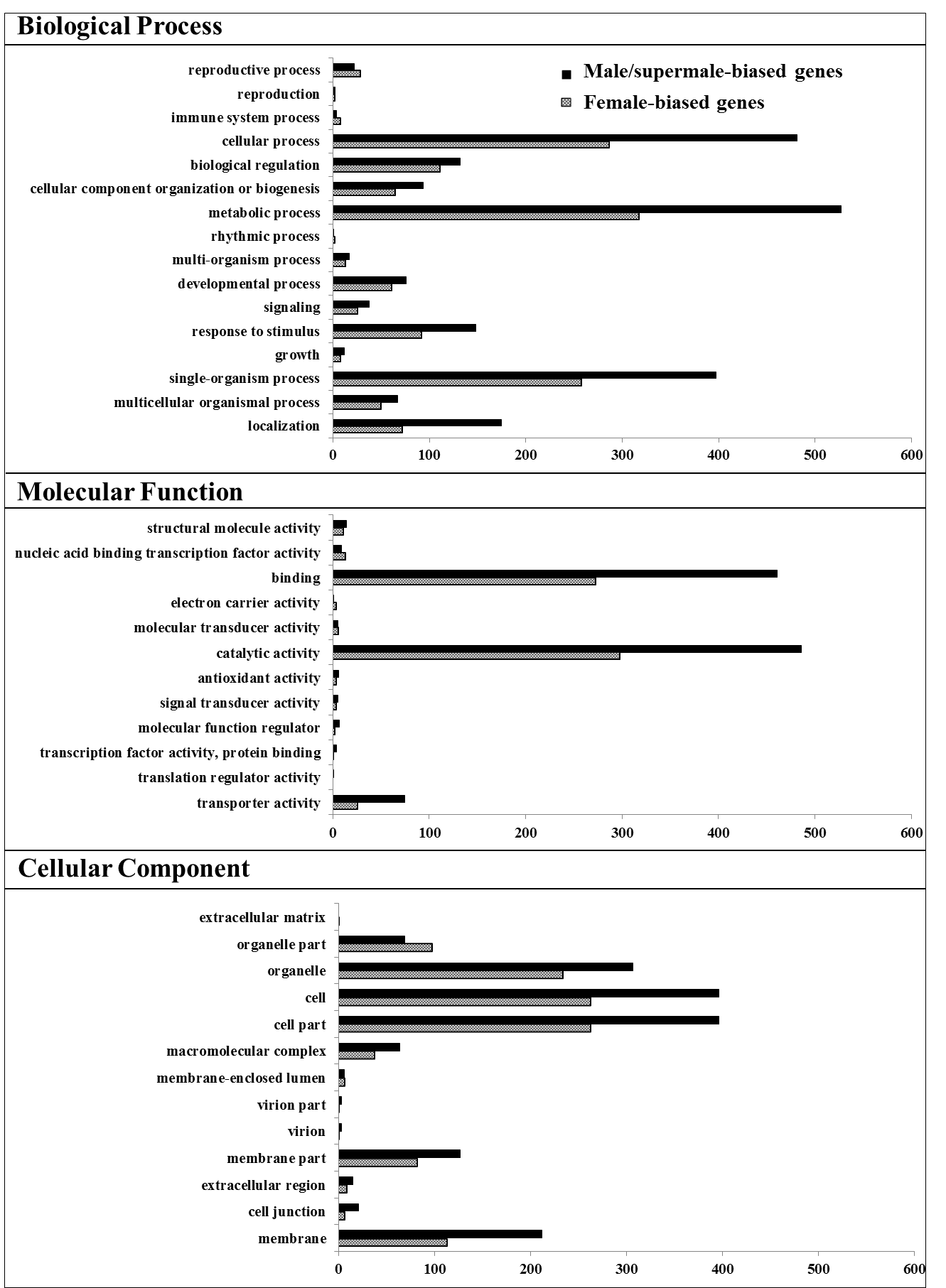

Supplement: Supplementary file 6 — Functional classification of DEGs in male/supermale and female flower buds. (TIFF 755 kb) [file 12870_2017_1091_MOESM6_ESM.tif]
